# Central Venous Catheter-Associated Superior Vena Cava Syndrome

**DOI:** 10.7759/cureus.37756

**Published:** 2023-04-18

**Authors:** Catarina Silva Araújo, Rui M Domingues, Peniela Couto, Ana Rita Matos, Cristina C Ângela

**Affiliations:** 1 Internal Medicine, Hospital de Braga, Braga, PRT; 2 Internal Medicine, Hospital Central de Maputo, Maputo, MOZ

**Keywords:** central venous catheter thrombosis, implantable venous port, venous stenting, superior vena cava thrombosis, superior vena cava syndrome

## Abstract

Superior vena cava syndrome (SVCS) is caused by any obstruction to the superior vena cava (SVC); the most common causes are malignancy and extrinsic compression. The use of medical devices, such as central venous catheters, poses an important risk factor, as they cause changes in the blood flow and in the vessel wall.

This report describes a case of a 70-year-old male with an implantable central venous port, due to previous neoplastic disease, as the cause of the SVCS.

Authors advise that the placement of medical devices ought to be carefully accessed and constantly revised to be removed when no longer needed to prevent avoidable complications.

## Introduction

Superior vena cava syndrome (SVCS) is caused by any obstruction or occlusion of the superior vena cava [1-3]. The most common causes are malignant obstruction (neoplastic invasion of the vein) or extrinsic compression, which causes damage in the vessel wall and a change in blood flow, potentiating thrombosis (i.e., lymph node engorgement and post-radiotherapy-associated fibrosis), although both mechanisms can coexist [1-4]. The incidence of device-related SVCS has been increasing due to the more frequent use of central venous catheters, pacemakers, and implantable defibrillators [3].

## Case presentation

A 70-year-old male, a retired carpenter, with a history of dyslipidemia was diagnosed with epidermoid carcinoma of the mouth seven years ago (pT4a G1 N0 R1). That same year, he underwent surgery and lymph node excision with surgical reconstruction, followed by chemotherapy and radiotherapy.

He was admitted several times to the emergency room (ER) for facial edema and redness, associated with dyspnea; edema of both arms, predominantly the left; and serous cutaneous exudate, which started three weeks prior and progressively worsened. At this point, he was in remission but still had the implantable jugular venous port.

**Figure 1:**
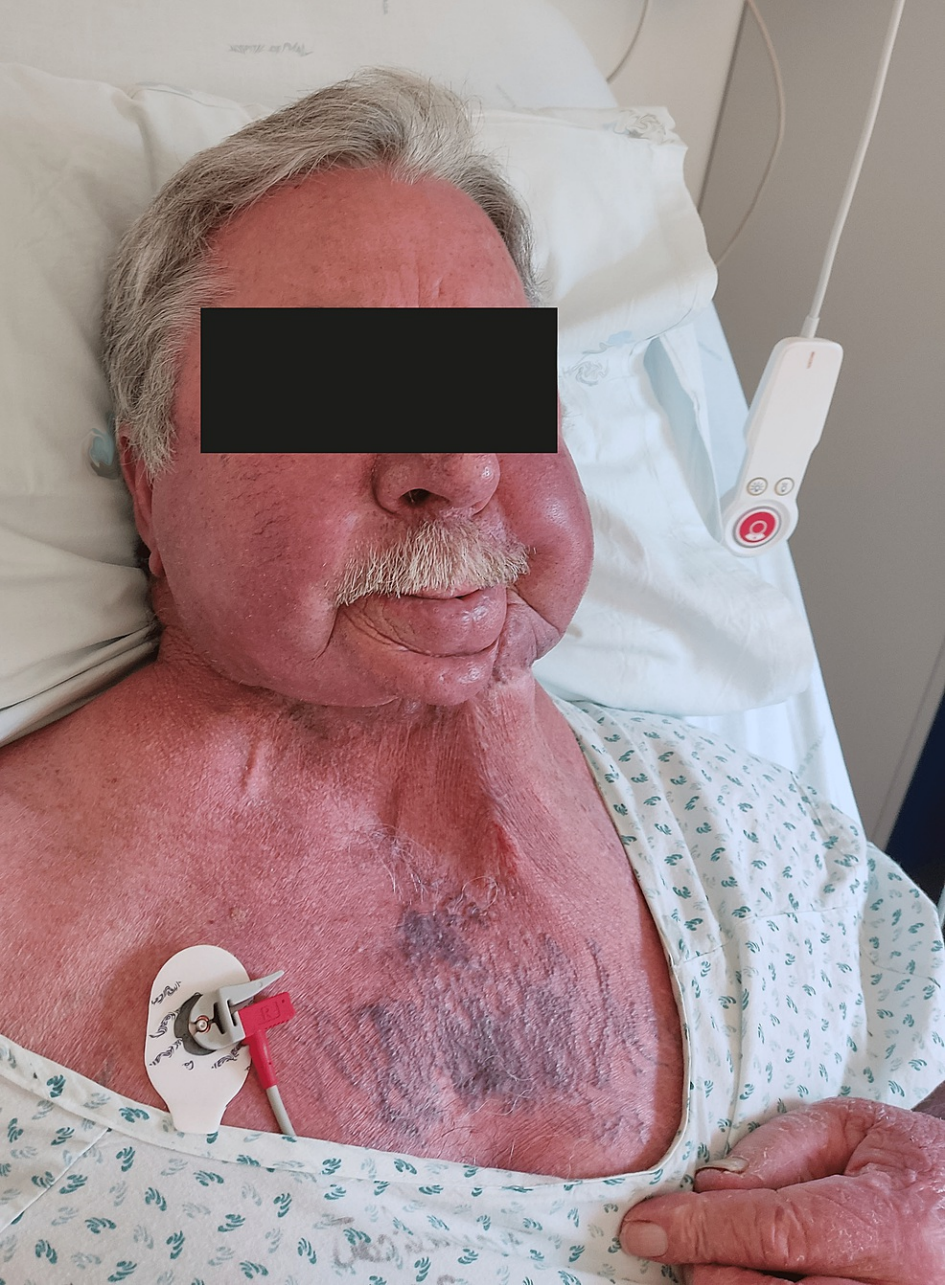
Photograph of the patient upon admission, before undergoing revascularization treatment, showing redness and swelling of the face, neck, and hand, along with notorious chest collateral circulation.

**Figure 2:**
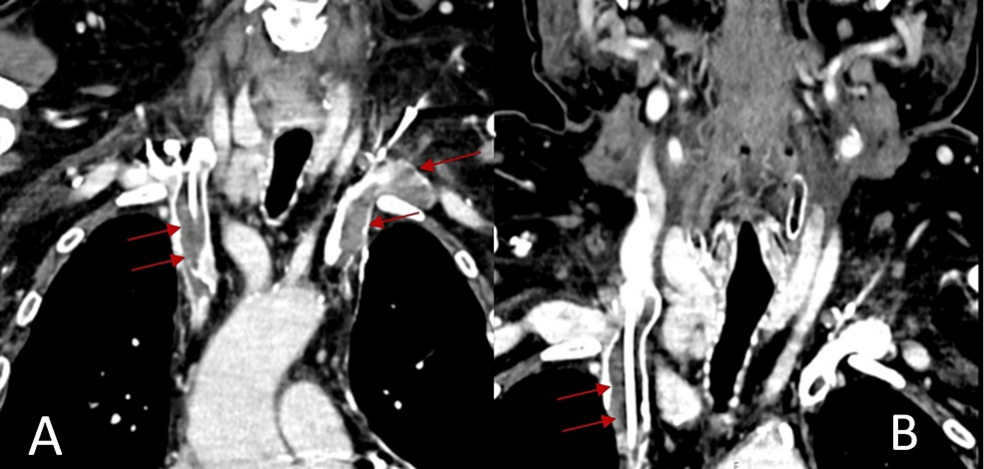
Thoracic (A) and cervical (B) angio-CT images showing thrombi in the superior vena cava and left subclavian and right internal jugular veins, as well as a right jugular central venous catheter (red arrows).

Initially, the patient was medicated with 20 mg/day of prednisolone because the considered diagnosis was allergic edema, and by the time of the last admission, it was increased to 60 mg/day, and pantoprazole and ebastine were added. The patient returned after a couple of days with the same symptoms: there was edema of the arms, predominantly on the left side, with erythematous lesions on the ipsilateral hand; erythema and edema of the face, lips, and tongue were also present (Figure 1); and collateral circulation in the pectoral region was noticed, as well as jugular venous engorgement. The patient presented with high blood pressure but was afebrile and had no respiratory distress. The blood work showed elevated leukocytes (11.2 × 10^3^/µL; normal range: 4-11 × 10^3^/µL) and D-dimer (1,063 ng/mL; normal range: <500 ng/mL), and hemoglobin, platelets, and renal function were normal. Chest X-ray and duplex ultrasound of the arms were normal. As such, a contrast CT of the neck and thorax was done (Figure 2), which confirmed the presence of venous catheter-associated thrombus in the right internal jugular vein and large thrombi in the right subclavian and external jugular vein, as well as in the superior vena cava. No signs of malignancy or enlarged lymph nodes were observed in the examination. The patient was seen by an otolaryngologist, who considered that there was no immediate risk of airway obstruction but recommended treatment with steroids to reduce upper airway edema. Subsequently, he was admitted to the internal medicine ward with the diagnosis of superior vena cava syndrome (SVCS). Low-molecular-weight heparin (1 mg/kg bid) and methylprednisolone (61.5 mg/daily) were initiated.

**Figure 3:**
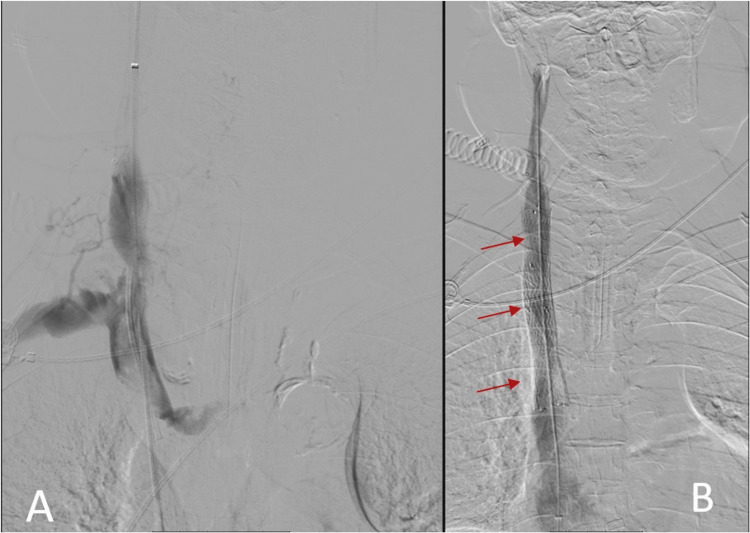
Angiography images before (A) and after (B) angioplasty with venous stenting (red arrows).

An endovascular procedure was done five days after admission, and after starting anticoagulation, balloon angioplasty was performed, followed by stent placement (Figure 3) and catheter removal, maintaining low-molecular-weight heparin until hospital discharge. No evidence of neoplastic relapse was found, and as there were no signs or past medical history that suggested a hypercoagulability state, the most likely cause for the SVCS was implantable venous port-associated thrombosis of the jugular vein. The patient was discharged asymptomatic and medicated with rivaroxaban 20 mg/daily with programmed re-evaluation in the outpatient clinic.

## Discussion

The diagnosis of SVCS is based on clinical signs and imaging [1,3,5]. Most patients present with altered chest radiography (mediastinal widening and pleural effusion reported in 64% and 26% of cases, respectively) [5]. Contrast-enhanced CT scan usually depicts the location and extent of the obstruction and can also determine the cause of obstruction, distinguishing thrombosis from extrinsic compression and benign from malignant causes and showing collateral circulation [1,3]. Duplex ultrasound, catheter-based digital subtraction venography, and magnetic resonance venography can also be informative [1].

Patients with neoplastic disease are at increased risk of developing this syndrome, as many have implantable central venous ports besides being in a hypercoagulability state [1-4]. The tell-tale sign of this syndrome is venous congestion of the head, neck, and upper extremities, to which the time of onset/disease progression and collateral vessel formation can worsen or mitigate, respectively, as was this case [4]. Dyspnea and neurological symptoms can also be present [3]. Acute mortality is rare, but clinicians must be aware of alarming signs, such as hemodynamic compromise, laryngeal edema, or signs of cerebral edema [3]. The incidence of SVCS associated with implantable devices is on the rise, as its use becomes ubiquitous, although extrinsic compression or invasion of the vessels are still the most relevant causes of SVCS [6].

Treatment of SVCS will depend on the cause of obstruction/thrombosis, usually decided by multidisciplinary teams. Treatment options can include chemo- or radiotherapy in the case of neoplastic disease, endovascular angioplasty or stenting for extrinsic compressions, catheter-based thrombus removal, or surgical bypass [1,3,7-9]. If thrombosis is present, anticoagulation should be started, but the duration of treatment is guided by clinical judgment [3]. Loop diuretic agents and parenteral steroids are frequently used, but evidence of efficacy is lacking [3,8,9].

Literature supporting the best time for the removal of implantable venous ports after cancer treatment is lacking, so future studies should address this problem. In this case, the removal of the venous port as soon as it was no longer needed would have prevented SVCS.

This patient presented with an insidious manifestation, which could be, in part, due to the presence of collateral circulation. The many thrombi in multiple vessels were most likely due to the delay in the diagnosis and therapy and are rarely described in the literature. Regardless, treatment was effective and resulted in complete and expeditious recovery. Medical devices, such as central venous catheters, are important risk factors for SVCS and are causing an increased incidence of this syndrome [3]. Their use should be limited according to the patient’s needs, being removed as soon as they are expected not to be used.

## Conclusions

Non-malignant SCVS are most commonly associated with intravascular devices, as was this case. This case reminds us to consider all diagnostic options, suspecting this diagnosis when typical signs start, avoiding diagnosis and treatment delay. The placement of medical devices ought to be carefully accessed, constantly revised, and removed when no longer needed.
